# The incidence of venous thromboembolism and practice of deep venous thrombosis prophylaxis in hospitalized cirrhotic patients

**DOI:** 10.1186/1477-9560-9-1

**Published:** 2011-01-18

**Authors:** Abdulaziz Aldawood, Yaseen Arabi, Abdulrahman Aljumah, Alawi Alsaadi, Asgar Rishu, Hasan Aldorzi, Saad Alqahtani, Mohammad Alsultan, Afaf Felemban

**Affiliations:** 1 Intensive Care Department, King Saud Bin Abdulaziz University for Health Science-King Abdulaziz Medical City, Riyadh, 11426, Saudi Arabia; 2 Hepatobiliary Science Department, King Saud Bin Abdulaziz University for Health Science-King Abdulaziz Medical City, Riyadh, 11426, Saudi Arabia; 3 Obstetric and Gynecology Department, King Saud Bin Abdulaziz University for Health Science-King Abdulaziz Medical City, Riyadh, 11426, Saudi Arabia

## Abstract

**Background:**

Cirrhotic patients are characterized by a decreased synthesis of coagulation and anticoagulation factors. The coagulopathy of cirrhotic patients is considered to be auto-anticoagulation. Our aim was to determine the incidence and predictors of venous thromboembolism (VTE) and examine the practice of deep venous thrombosis (DVT) prophylaxis among hospitalized cirrhotic patients.

**Methods:**

A retrospective cohort study was performed in a tertiary teaching hospital. We included all adult patients admitted to the hospital with a diagnosis of liver cirrhosis from January 1, 2009 to December 31, 2009. We grouped our cohort patients in two groups, cirrhotic patients without VTE and cirrhotic with VTE.

**Results:**

Over one year, we included 226 cirrhotic patients, and the characteristics of both groups were similar regarding their clinical and laboratory parameters and their outcomes. Six patients (2.7%) developed VTE, and all of the VTEs were DVT. Hepatitis C was the most common (51%) underlying cause of liver cirrhosis, followed by hepatitis B (22%); 76% of the cirrhotic patients received neither pharmacological nor mechanical DVT prophylaxis.

**Conclusion:**

Cirrhotic patients are at risk for developing VTE. The utilization of DVT prophylaxis was suboptimal.

## Introduction

Liver cirrhosis is a major health problem worldwide, especially in Saudi Arabia[1] where the prevalence of liver cirrhosis is not precisely known but is expected to be high due to the relatively high prevalence of viral hepatitis[2-4]. Liver cirrhosis is accompanied by multiple changes in the hemostatic system due to the reduced levels of natural inhibitors of coagulation and coagulation factors because of the impaired hepatic synthetic activity [5]. Thus, the global effect of liver disease on hemostasis is complex, and therefore, patients with liver cirrhosis can experience bleeding or thrombotic complications [6]. The pathogenesis of venous thromboembolism (VTE) in cirrhosis is complex and involves several factors, both endogenous changes associated with cirrhosis with increased levels of factors VII and also protein C activity is limited in the absence of the endothelial receptor thrombomodulin and therefore it cannot exert its full anti-coagulant activity as well as external factors, one of which is limited physical activity due to the disease itself [7-10]. The incidence of VTE among high-risk hospitalized patients has been reported to range between 4 and 12%[7]. In a large necropsy series, in which fatal VTE accounted for 7-10% of all hospital-related deaths, 70-90% of the patients had no premorbid symptoms [11]. Among these subjects with VTE, approximately 25% died within 7 days of VTE onset[12]. In addition, the American College of Chest Physicians (ACCP) guidelines on VTE prophylaxis do not specifically address patients with coagulopathy due to liver cirrhosis [13]. Based on the presence of coagulopathy in cirrhotic patients, these patients are considered to be auto-anticoagulant[14]. Hence, the institution of deep vein thrombosis (DVT) prophylaxis in cirrhotic patients may not be a standard practice, and the use of DVT prophylaxis in this patient population is expected to be variable.

The objective of the present study was to determine the incidence and predictors of VTE and to examine the practice of DVT prophylaxis among hospitalized cirrhotic patients.

## Methods

### Study population

A retrospective chart review was conducted of patients with discharge ICD-9 diagnosis codes corresponding to liver cirrhosis who were admitted to a tertiary care hospital in Riyadh, Saudi Arabia, from January 1, 2009 to December 31, 2009. These patients were adults of 18 years or older and had a history and clinical presentation consistent with liver cirrhosis and/or a liver biopsy showing cirrhosis. Patients on anticoagulation therapy were excluded from the study. The study was approved by King Abdullah International Medical Research Center and the institutional review board of the hospital (IRB). The approval allowed for a retrospective chart review without informed consent.

### Data collection

For each patient, the following information was collected: age, gender, admission creatinine, international normalized ratio (INR), bilirubin, albumin and platelet counts, etiology of liver cirrhosis (viral hepatitis, alcohol, autoimmune, cryptogenic), Child-Pugh score, VTE risk factors (history of malignancy, prior VTE, hormonal replacement therapy, oral contraceptives), use of pharmacologic prophylaxis in the form of unfractionated heparin (UFH) or low molecular-weight heparin (LMWH) and the use of mechanical prophylaxis. Patients were followed up until discharge from the hospital or until death, whichever was earlier.

### Outcome measures

The primary outcome was defined as the development of symptomatic DVT or PE, as confirmed by venous Doppler ultrasound (VD-US) of the lower limbs, spiral CT of the chest or a high probability ventilation-perfusion (VQ) scan. These tests were ordered by the treating physician based on clinical suspicion. The secondary outcome was the use of DVT prophylaxis.

### Statistical analysis

Continuous data were compared using the Kruskal-Wallis test and are expressed as the median and Interquartile range according to normality testing using Kolmogorov-Smirnov test. Categorical data were compared using the chi-square test or Fisher's exact test and are expressed as a percentage. Statistical significance was defined as alpha less than 0.05. Statistical analysis was performed using Minitab for Windows (release 13.1).

## Results

### General characteristics

During the study period, 226 patients were admitted to the hospital with liver cirrhosis. Only six (2.7%) patients were diagnosed with VTE. Table 1 summarizes the characteristics of the cirrhotic patients without VTE (n = 220) and with VTE (n = 6). Both groups were similar with respect to baseline characteristics, including admission albumin, bilirubin, creatinine and coagulation profile. Hepatitis C accounted for 115 (51%) of the liver cirrhosis cases, followed by hepatitis B accounting for 49 cases (22%) and uncommon causes such as cryptogenic, autoimmune or alcohol. However, the etiology of liver cirrhosis and the prevalence of hepatocellular carcinoma were similar in both groups. Infection was the most common cause of hospital admission in this group of patients (53.5%), followed by bleeding (23.8%). The need for intensive care unit(ICU) admission was similar in both groups. There was a trend toward a higher Child-Pugh score in cirrhotic patients with VTE.

**Table 1:** Characteristics of patients with liver cirrhosis

Characteristic	Cirrhotic patients with VTE	Cirrhotic patients without VTE	*P *value
Number (%)	6 (2.7%)	220 (97.3%)	

Age in years*,	63.5(49-83.7)	63(54-70)	0.67

Sex			0.57
Male, N (%)	2 (33.3%)	138 (62.7%)	
Female, N (%)	4 (66.6%)	82 (37.3%)	

INR*	1.55(1.25-2.15)	1.3(1.1-1.6)	0.24

Patients admitted with INR≥ 1.8	1(16.6%)	40(18.2%)	1

APTT*	36.75 (29.3-38)	33(29-40)	0.75

Platelet count/μL*	201(122-265)	136(81-206)	0.12

Albumin (g/L)*	26.5(23.5-32.2)	30(26-35)	0.23

Bilirubin (μmol/L)*	28(14.7-209.4)	37.4(18-85.5)	0.88

Creatinine (μmol/L)*	93(87.5-138.6)	81(73-151)	0.49

Etiology of liver cirrhosis Hepatitis B N (%)	2 (33.3%)	47 (21.3%)	0.6

Hepatitis C N (%)	3 (50.0%)	112 (50.9%)	1

Alcoholic N (%)	0	0	

Autoimmune N (%)	0	4 (1.8%)	

Cryptogenic N (%)	1 (16.6%)	46 (20.9%)	

Other N (%)	0	16(7%)	

Patients admitted with Infections	2 (33%)	119(54%)	0.42

Bleeding	2(33%)	52(23.6%)	0.63

Hepatocellular carcinoma N (%)	1 (16.6%)	100 (45.5%)	0.22

Incidence of ICU admission N (%)	2 (33.3%)	45 (20.5%)	0.6

Child-Pugh stage, mean ± SD	10.3 ± 1.97	8.25 ± 2.57	0.052

Grade A N (%)	0	70 (31.8%)	

Grade B N (%)	3 (50%)	84 (38.2%)	0.68

Grade C N (%)	3 (50%)	72 (32.7%)	0.4

Hospital LOS*,	43(12.3-75.5)	8(4.25-14.7)	0.004

Hospital mortality N (%)	4 (66.6%)	73 (33.2%)	0.18

* Expressed as Median (Interquartile range)

INR: international normalized ratio

APPT: activated partial thromboplastin time

LOS: length of stay

### Incidence and risk factors of VTE

Six patients (2.7%) developed DVT, which was identified clinically and confirmed by VD-US. None of the cirrhotic patients developed pulmonary embolism; the spiral CT of the chest was done only once and turned to be negative. There was no statistically significant difference in the incidence of VTE among the two groups based on the reason of the admission to the hospital.

DVT was suspected in 18 (8%) cirrhotic patients and was confirmed only in 6 (2.7%) patients. A previous history of VTE was higher in cirrhotic patients with VTE (0 vs. 2), whereas the other VTE risks factors were similar in the two groups (Table 2).

**Table 2:** Risk factors for venous thromboembolism (VTE) among cirrhotic patients

VTE risk factors	Cirrhotic patients with VTE N = 6	Cirrhotic patients without VTE N = 220	*P *value
History of DVT	2 (33.3%)	0 (0%)	

Post-operative	0	6 (2.7%)	

Bedridden	0	7 (3.2%)	

History of malignancy	0	5 (2.3%)	

Femoral central line	1 (16.7%)	30 (13.6%)	0.57

IJ &SC central line	1 (16.7%)	35 (15.9%)	1

Diabetes mellitus	4 (66.7%)	91 (41.3%)	0.24

Values are expressed as numbers and percentages

IJ: internal jugular, SC: subclavian

### Practices of VTE prophylaxis

Approximately 76% of the cirrhotic patients included in the cohort received neither pharmacological nor mechanical DVT prophylaxis (Table 3). Only four cirrhotic patients without VTE (1.8%) received LMWH. No significant differences in the incidence of VTE were observed between the group that received pharmacologic or mechanical prophylaxis and the group that did not receive prophylaxis. Further, we carried out subgroup analysis and found that there was no difference in the use of prophylaxis in patients admitted due to infection, HCC and patients with high INR (>1.8) except mechanical prophylaxis was used more in patients admitted due to bleeding (P value 0.01).(table 3).

**Table 3:** The impact of VTE prophylaxis on the incidence of VTE in all hospitalized cirrhotic patients and subgroups of hospitalized cirrhotic patients

DVT prophylaxis	VTE 6	No VTE 220	OR(95%CI)	P value
All Patients				
Pharmacologic	1/6 (16.7%)	26/220 (11.8%)	1.49 (0.16-13.2)	0.54
Mechanical	2/6 (33.3%)	25/220 (11.9%)	3.9 (0.68-22.4)	0.15
None	3/6 (50%)	169/220 (78%)	0.28 (0.08-1.6)	0.13

Patients admitted with infections				
Pharmacologic	1/2 (50%)	15/119(12.6%)	6.9 (0.41-116)	0.25
Mechanical	0/2(0%)	15/119 (12.6)	0.98 (0.96-100)	0.76
None	1/2 (50%)	89/119(74.8%)	0.31 (0.19-5.1)	0.42

Patients admitted with Bleeding				
Pharmacologic	0/2(0%)	7/52 (13.5%)	0.96(0.9-1.02)	0.76
Mechanical	2/2(100%)	4/52 (7.71)	1.5 (0.85-2.6)	0.01
None	0/2(0%)	41/52(7.8-8.8%)	NA	0.06

Patients with HCC				
Pharmacologic	0/1(0%)	11/101 (10.9)	NA	0.89
Mechanical	1/1(100%)	8/101(7.9%)	1.1 (2.9-1.4)	0.09
None	0/1(0%)	82/101(81%)	NA	0.19

Patients with INR ≥ 1.8				
Pharmacologic	0/1(0%)	0/1(0%)	0.98(0.93-1..03)	0.95
Mechanical	1/1 (100%)	7/40 (17.5%)	1.14(0.88-1.149)	0.19
None	0/1(0%)	31/40(77%)	0.89(0.7-1.12)	0.2

HCC: Hepatocellular carcinoma

INR: international normalized ratio

## Discussion

The incidence of VTE was 2.7% in hospitalized cirrhotic patients, where the VTE evaluations were performed based on clinical suspicion. The majority of patients (76.1%) received neither pharmacological nor mechanical DVT prophylaxis. The underlying coagulopathy observed in cirrhotic patients has led to the notion that these patients might be at a lower risk for VTE [7,15]. This hypothesis was suggested in a population-based case-control study[12] performed in the last century, which found that serious liver disease was associated with a 90% reduction in the risk for VTE. However, that study lacked the data on the severity of the liver diseases, use of DVT prophylaxis, and the diagnosis of VTE was not performed using the standard practices [12]. However, the specific insight is inconsistent with the results of many studies that have addressed the incidence of VTE in cirrhotic patients. Recently a Danish nationwide population- base case- control study [9] showed that the liver cirrhosis was associated with increased relative risk of VTE; 1.74 (95% CI,1.54-1.95) and in patients with unprovoked VTE, relative risk was slightly higher; 2.06(95% CI,1.79-2.38, regardless of the presence of other risk factors. A case-control study[16] showed that the prevalence of DVT in cirrhotic patients was 4.7% and that diabetes mellitus was an independent risk factor for the development of DVT, which were not found in the present study. Northup et al. [17]conducted a retrospective cohort study over an 8-year period and determined an incidence of VTE among 21,000 cirrhotic patients (in whom alcohol was the most frequent underlying etiology) of 0.5%, which had lower incidence of VTE (4 to 12%) found in selected subgroups of patients at the same institution. They also found that a low serum albumin level was an independent predictor of VTE in cirrhotic patients and that 79% of cirrhotic patients received neither pharmacological nor mechanical DVT prophylaxis, which is similar to the rate observed in our study. Another case-control study by Gulley et al. [7] found 1.8% incidence of VTE and the risk for VTE was not lower than that determined for matched non-cirrhotic controls without selected co-morbidities, a finding similar to our study. Dabbagh et al[14]. found an incidence of VTE of 6.3% in cirrhotic patients, and although the utilization of DVT prophylaxis was suboptimal, there was no association between the incidence of VTE and prophylaxis. The high incidence of VTE could be explained by the greater morbidity of these patients, as reflected by their high child-Pugh scores. The failure of DVT prophylaxis should be interpreted with caution, because the low VTE incidence and low rate of DVT prophylaxis utilization preclude the establishment of firm conclusions. Our result and others [7,16,17] showed that the incidence of VTE was not lower than that determined in matched non-cirrhotic patients, even in patients with cirrhosis of different etiologies and with varying levels of severity.

Several mechanisms have been proposed to explain the observed thrombosis in cirrhotic patients. An acquired deficiency of antithrombotic III, protein C and protein S and the presence of antiphospholipid antibodies are observed in patients with cirrhosis [5,18,19]. In addition to a decreased synthesis of anticoagulants, cirrhotic patients are prone to hypercoagulation due to a chronic inflammatory state that results in poor flow and vasculopathy [16,20]. Lisman et al[21] found thrombin generation is equal or superior in patients with liver cirrhosis undergoing liver transplantation compared to healthy volunteers in the presence of exogenous thrombomodulin. An imbalance between the hemostatic process, thrombosis may produce a prothrombotic state in which the risk of thrombosis is increased [22]. Our study has several limitations that are unavoidable in a retrospective study. It was conducted in one center where the sample size was relatively small. In addition, some of the variables extracted from the medical charts (such as patient lifestyle) were not available, which could confound our results as well as the suspecting PE is very low as reflected by the fact that only one spiral CT of chest was done. Despite these potential weaknesses, few studies have addressed this issue, and there are no guidelines regarding DVT prophylaxis in hospitalized cirrhotic patients. The present study elucidates possible predictors of the risk for VTE in this group of patients and may aid in determining the patients who will benefit from DVT prophylaxis, despite the notion that auto-anticoagulation protects against VTE in cirrhotic patients[17]. These data will pave the way for further studies designed to evaluate the role of DVT prophylaxis in cirrhotic patients and alert healthcare providers to consider VTE in the differential diagnosis of cirrhotic patients with coagulopathy when these patients present with clinical features that are compatible with VTE.

Until the risks and benefits of VTE prophylaxis are established in this particular population, the VTE prophylaxis can not be withdrawn in the cirrhotic population at present time[23].

In conclusion, the incidence of VTE in hospitalized cirrhotic patients was 2.7%, and the utilization of DVT prophylaxis was suboptimal. Because the treatment and prophylaxis for VTE carries an increased risk of bleeding, additional prospective multicenter studies should be conducted to address the benefits and risks of this intervention.

## Competing interests

The authors declare that they have no competing interests.

## Authors' contributions

AAL: carried out the design, interpretation, statistical analysis, drafting of the manuscript and critical revision of the manuscript

YA: carried out the design, statistical analysis and critical revision of the manuscript

AJ: carried out the design, interpretation, and drafting of the manuscript

AA: carried out the data collection, the design, interpretation and drafting of the manuscript

AR: carried out the data collection, the design, statistical analysis, interpretation and drafting of the manuscript

HD: carried out the design, interpretation and critical revision of the manuscript

SQ: carried out the design, statistical analysis and critical revision of the manuscript

MS: carried out the design, statistical analysis and critical revision of the manuscript

AF: carried out the design, statistical analysis and critical revision of the manuscript

All authors read and approved the final manuscript
